# Inhibition of 5′-UTR RNA Conformational Switching in HIV-1 Using Antisense PNAs

**DOI:** 10.1371/journal.pone.0049310

**Published:** 2012-11-12

**Authors:** Braham Parkash, Atul Ranjan, Vinod Tiwari, Sharad Kumar Gupta, Navrinder Kaur, Vibha Tandon

**Affiliations:** 1 Dr. B. R. Ambedkar Center for Biomedical Research, University of Delhi, Delhi, India; 2 Department of Chemistry, University of Delhi, Delhi, India; Institut National de la Santé et de la Recherche Médicale, France

## Abstract

**Background:**

The genome of retroviruses, including HIV-1, is packaged as two homologous (+) strand RNA molecules, noncovalently associated close to their 5′-end in a region called dimer linkage structure (DLS). Retroviral HIV-1 genomic RNAs dimerize through complex interactions between dimerization initiation sites (DIS) within the (5′-UTR). Dimer formation is prevented by so calledLong Distance Interaction (LDI) conformation, whereas Branched Multiple Hairpin (BMH) conformation leads to spontaneous dimerization.

**Methods and Results:**

We evaluated the role of SL1 (DIS), PolyA Hairpin signal and a long distance U5-AUG interaction by *in-vitro* dimerization, conformer assay and coupled dimerization and template-switching assays using antisense PNAs. Our data suggests evidence that PNAs targeted against SL1 produced severe inhibitory effect on dimerization and template-switching processes while PNAs targeted against U5 region do not show significant effect on dimerization and template switching, while PNAs targeted against AUG region showed strong inhibition of dimerization and template switching processes.

**Conclusions:**

Our results demonstrate that PNA can be used successfully as an antisense to inhibit dimerization and template switching process in HIV -1 and both of the processes are closely linked to each other. Different PNA oligomers have ability of switching between two thermodynamically stable forms. PNA targeted against DIS and SL1 switch, LDI conformer to more dimerization friendly BMH form. PNAs targeted against PolyA haipin configuration did not show a significant change in dimerization and template switching process. The PNA oligomer directed against the AUG strand of U5-AUG duplex structure also showed a significant reduction in RNA dimerization as well as template- switching efficiency.The antisense PNA oligomers can be used to regulate the shift in the LDI/BMH equilibrium.

## Introduction

Electron microscopy of packaged HIV-1 genomic RNAs revealed that the two RNA molecules are strongly associated with each other through their 5′ ends, which are termed as dimer linkage structure [1], [2]. Various studies show that the diploid viral genome provides advantages over the monomeric form – (i) dimeric RNA is preferentially packaged over monomeric RNA [3], [4]. (ii) The dimeric form provides the opportunity for switching from a damaged template to a physically linked intact template during reverse transcription. (iii) Dimers also facilitate recombination through template switching, which causes an increase in the genetic diversity and viral adaptability [5].

**Figure 1:**
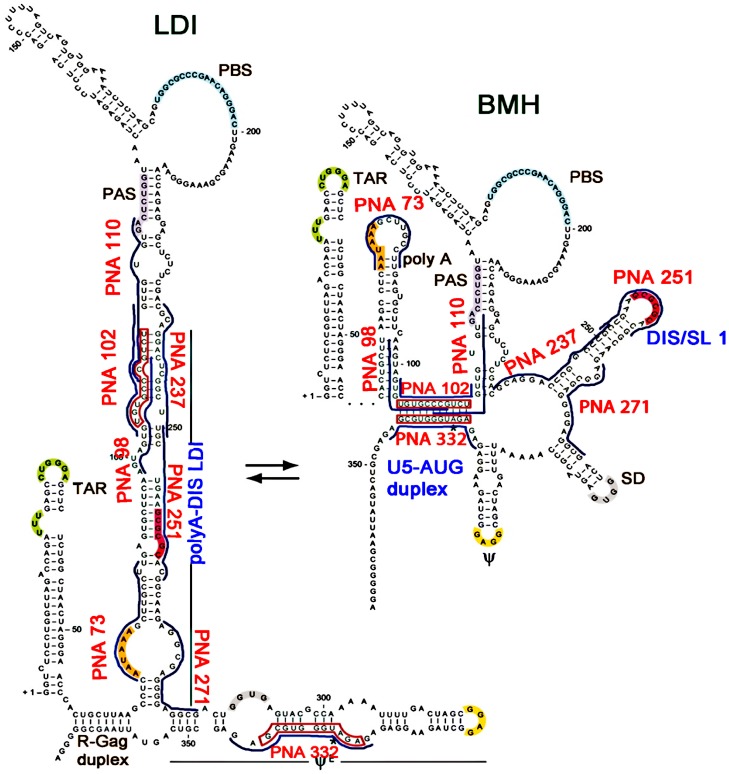
Proposed stem-loop structure of the 5′untranslated region of HIV-1. Target loop regions are highlighted. PNAs targeted against different sites of 5′UTR of HIV-1 are shown in the diagram (Abbink *et. al.* 2003).

Secondary structure of 5′-UTR of HIV-1 genomic RNA lead a riboswitch model in which extensive structural rearrangements of leader sequence could influence the presentation of stem-loop 1 (SL1), polyA hairpin, other RNA signals and thus dimerization, and template switching efficiency [6]–[12]. Later on it was proved that full length 5′-UTR adopts thermodynamically stable long-distance interaction (LDI) conformation which can shift to metastable branched multiple hairpin conformation (BMH). The downstream region including the gag start codon folds differentially in the LDI and BMH structures (Fig. 1, 2A) [13]. Paillart *et. al.* proposed the existence of various stem loop motifs and the genomic RNA associated with infected cells in the dimerization and packaging competent BMH form, and they argued against the existence of the LDI conformer [14] The BMH contains exposed sequences within the trans-activation region (TAR), polyadenylation signal hairpin (poly A), primer binding site (PBS) domain, dimerization initiation site (DIS or SL1), and the U5-AUG duplex between a C-rich sequence in the 5′part of U5 and a G-rich sequence around the *gag* translation initiation region. The U5-AUG and SL1 regions appear to play an important role in dimerization [15]. HIV-1 genomic RNA (gRNA) dimerization is mainly attributed to dimerization site located in SL1, also named as dimerization initiation site (DIS) [16]–[18].This is involved in gRNA dimerization involving “kissing” mechanism forming duplex between two DIS [19], [20]. The U5-AUG duplex helps in gRNA dimerization through displacing and exposing the DIS. The role of TAR could be direct, via TAR-TAR kissing interaction, or indirect, via an effect on 3′ sequences excluding SL1. Jalalirad.et. al. (2012) provided evidence that the TAR bulge and upper TAR stem-loop contribute to HIV-1 gRNA dimerization, but not by a mechanism that involves TAR-TAR kissing [21], [22].

The retroviruses can use template switching to repair a break or damage in the RNA templates, a process known as forced copy-choice recombination [23]. The study with various HIV-1 leader mutants showed a correlation between the status of the LDI-BMH equilibrium, dimerization and packaging of viral RNA [12], [24]. Several studies of mutant sequences indicated that the LDI/BMH conformation of HIV-1 leader sequence has no direct effect on mRNA translation or reverse transcription initiation [24], [25], Destabilization of the TAR hairpin even by single mutation affects the LDI/BMH confirmation equilibrium and function of the HIV-1 leader RNA [26], [27]. A high-throughput SHAPE analysis and a revisit to architecture and secondary structure of full length HIV-1 RNA genome suggested that RNA encodes not only for protein primary sequences, but it also modulates ribosome elongation to promote native protein folding [28], [29].

Dimerization of retroviral RNA is a prerequisite to viral encapsidation/packaging [30], [31]. Jun-IchiSakuragi *et.al.* found that most of the U5/L stem-loop is not required, only 10 bases within the lower SL1 stem-loop is required for complete RNA dimerization [32]. Consistent with this finding, Harrison et al. (1998) reported that SL1 stem corruption or loop deletion causes partial packaging and replication defects [8]. Deletion or mutation in the SL1 prevents RNA dimerization and lead to a marked decrease in infectivity and packaging of the viral genome. Deletion in DIS significantly inhibits plus-strand DNA synthesis which is associated with second-strand transfer level in the process of strand transfer. Thus this proviral DNA along with other recognition site for a viral factor is necessary for efficient strand transfer and dimerization [15], [33]–[35]. However, studies in viruses produced by tissue culture cells or peripheral blood mononuclear cells indicated that SL1 was not essential for gRNA dimerization [35]–[39]. However, Balakrishnan *et al.* suggested that transfer efficiency was severely compromised upon deletion of the DIS hairpin region from the UTR sequence and was enhanced when this element was inserted within a non-UTR sequence [40]. It has also been reported that a mutation in the DIS/DLS site did not affect dimer stability *in vivo* and that a region far from the DIS/DLS affected dimer formation in the retrovirus genome. Thus, there are many discrepancies between the *in vitro* and *in vivo* data on HIV-1 RNA dimerization [32], [41], [42].

Antisense and sense oligonucleotide have been previously been shown to inhibit HIV-1 RNA dimerization [43], [44]. Antisense oligonucleotides can shift the equilibrium between LDI and BMH conformations [45] and thus may affect various replication processes. However, one of the major limitations of the use of phosphodiester or phosphorothioate (e.g.; GEM 91) oligonucleotides in cells is their rapid degradation by nucleases [46]. We evaluated design and efficacy of antisense Peptide Nucleic Acids (PNA) targeted against the 5′-UTR, SL1 and polyA Hairpin part of HIV-1. PNAs bind with higher affinity and specificity to complementary nucleic acids (DNA or RNA) than their natural counterparts and obey the Watson-Crick base-pairing rules [47]–[49]. PNAs are not hydrolyzed by nucleases or proteases and this provides stability in biological fluids [50].

In the present study for the first PNAs were designed and synthesized in order to elucidate the importance of SL1, U5-AUG and PolyA Hairpin in dimerization and template-switching processes and the effect of these PNAs on the equilibrium between two alternative conformations. Our data reveal the importance of SL1 and AUG region as a potential target for antisense PNA as we observed strong inhibitory effect on dimerization and template-switching processes, whereas PNAs targeted against U5 region and PolyA hairpin signal showed little or no inhibition. Based on the results of our study, we suggest that the interference of HIV-1 leader RNA dimerization and template switching processes can be augmented by the use of antisense PNAs.

## Materials and Methods

### Chemicals and Reagents

All antisense PNA oligomers listed in Table 1 were synthesized manually, desalted by Sephadex G-25 (Amersham), purified by RP-HPLC. The antiparallel RNA sequences complementary to respective PNA were obtained from Eurogenetec, whereas DNA oligonucleotides were procured from Sigma. All primers were synthesized by Microsynth. The pYU-2 (5′) plasmid was generated by removing a ∼3.5 kb fragment from 3′ end of the HIV-1 genome from parent pYU-2 plasmid (# Acc. No- M93258, NIH-AIDS reagent program) with PacI and SphI restriction enzymes, was kindly provided by Dr. Uday Ranga (JNCSAR, Bangalore). Taq DNA polymerase was obtained from Bangalore Genei. dNTPs, T4 polynucleotide kinase and restriction enzymes were from MBI Fermentas. HIV-1 RT, RNase inhibitor, RNase-free DNase I were purchased from Ambion Inc. All other chemicals were purchased from Sigma Chemicals, St Louis, MO.

**Table 1:** Name and sequence of Peptide Nucleic Acid (PNA) targeted against different sites of 5′UTR HIV-1.

Name of PNA	Sequences of PNA (N C)	Position at HIV-1 RNA genome
PNA73	Lys-AAGGCAAGCTTTATT	73–87
PNA98	Lys-CGGGCACACACTACT	98–112
PNA102	Lys-CAGACGGGCACACAC	102–116
PNA110	Lys-TCACACAACAGACGG	110–125
PNA237	Lys-CAAGCCGAGTCCTGC	237–251
PNA251	Lys-CGTGCGCGCTTCAGC	251–265
PNA271	Lys-CGCCTCCCCTCGCCT	271–285
PNA332PNAscb	Lys-CTCGCACCCATCTTCLys-GCCCTTGCTCACCAT	332–346–

### UV T_m_ Measurements

The concentrations of PNAs were calculated on the basis of absorbance from the molar extinction coefficient of the nucleobases. The antiparallel PNA: RNA and PNA: DNA duplexes were prepared in 10 mM sodium phosphate buffer, pH 7 containing NaCl (100 mM) and EDTA (0.1 mM) and annealed by keeping the samples at 95°C for 5 min and followed by slow cooling to room temperature (annealing). Absorbance versus temperature profiles were obtained by monitoring at 260 nm with a UV-vis spectrophotometer scanning from 25 to 90°C temperature at a ramp rate of 0.2°C/minute. The measurements were performed in a 1-cm path length quartz cuvette containing PNA: RNA duplex at 2.5 µm concentration. Similar experiments were performed with PNA: DNA duplex to compare affinity of PNA with DNA. The melting studies were done on Cary 300 UV-Visible Spectrophotometer.

### Synthesis of RNA Template

The DNA template (474 bp) for transcription was made by PCR amplification of the plasmid pYU-2 (5′) using upstream primer T7F (5′ GGA TCC TAA TAC GAC TCA CTA TAG GGA GGT CTC TCT GGT TAG ACC A 3′) and downstream primer GR (5′ GCC CAT ACT ATA TGT TTT AAT 3′), containing a T7 promoter directly upstream of the +1 position of the wild type HIV-1 transcript and ends to the 5′ end of the *gag* gene (position +1 to 447 relative to the transcription start site +1). PCR reaction was separated on 1% agarose gel and purified using the QIAEX II Gel Extraction Kit (Qiagen). *In vitro* transcription was carried out using MAXI script T7 transcription kit (Ambion). The radiolabeled transcripts were synthesized by using (α-^32^P) UTP (1µl) (BARC India) and were purified on 4% denaturing TBE polyacrylamide gels. The RNA bands were excised and quantified by either UV-absorbance measurements or scintillation counting.

### Synthesis of Donor and Acceptor Constructs

**Figure 2:**
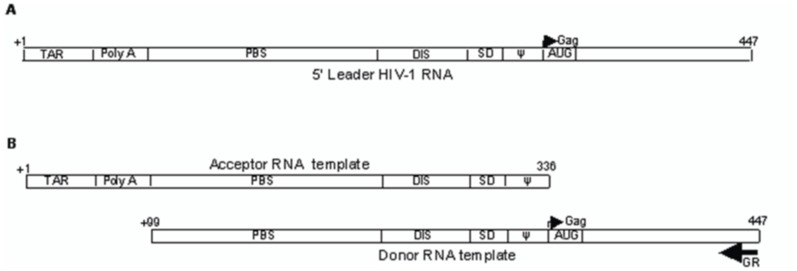
Schematic representation of RNA transcripts showing motifs within the 5′ UTR of HIV-1 RNA genome. (A)5′ leader HIV-1 RNA transcripts including the 5' end of the *gag* gene (position +1 to 447 relative to the transcriptional start site +1). (B) Acceptor and donor RNA templates used in the template-switching assays.

Acceptor constructs were generated by PCR amplification of the 335nt long region (positions +1 to 336 relative to the transcriptional start site, +1) of 5′UTR of HIV-1 using sense primer T7F (5′ GGA TCC *TAA TAC GAC TCA CTA TAG GGA* GGT CTC TCT GGT TAG ACC A 3′) and antisense primer T7R (5′ CTC TCT CCT TCT AGC CTC CGC 3′) in Taq Buffer (10 mM Tris-HCl (pH 9), 1.5 mM MgCl_2_, 50 mM KCl and 0.01% gelatin) under the following temperature conditions – (94°C - 4 min)×1, (94°C for 1 min, 60°C for 30 sec, 72°C for 1 min)×40, (72°C for 10 min)×1 and finally at 4°C for ∞. Donor construct was prepared by PCR amplification of the +90−447 sub-region (starts from +90 to 447 relative to the transcription start site, +1) of 5′UTR including *gag* gene of HIV-1using the primer set BR (5′ GTG CTT TAA GTA GTG TGT GCC 3′) and GR (5′ GCC CAT ACT ATA TGT TTT AAT 3′) (Fig. 2B) in Taq buffer under the following temperature conditions – (94°C for 4 min)×1, (94°C for 1 min, 47°C for 30 sec, 72°C for 1 min)×40, (72°C for 10 min)×1 and finally maintained at 4°C for ∞. After purification through the QIAEX II Gel Extraction Kit, the DNA fragment was cloned in pGEM-T easy vector (Promega). After finding the correct orientation of inserted fragment in pGEM-T easy vector, the final donor DNA template for transcription was generated by PCR amplification using upstream primer M13F and downstream primer GR in Taq buffer under the following temperature conditions - (94°C for 4 min)×1, (94°C for 1 min, 47°C for 30 sec, 72°C for 1 min)×40, (72°C for 10 min)×1 and then at 4°C for ∞. The donor and acceptor DNA templates were purified and were used for synthesis of RNA transcripts through *in-vitro* transcription as described in previous section. The acceptor and donor constructs were verified by sequencing.

### *In-vitro* Dimerization Assays

Dimerization assays were performed using a radiolabeled RNA template concentration of ∼2pmol. The RNA template was first incubated at 65°C for 10 min and then at 37°C for 30 min in 10 µl of dimerization buffer (50 mM Na-cacodylate, pH 7.5, 250 mM KCl, 5 mM MgCl_2_) with varying concentration of antisense PNA oligomers and the samples were then analyzed on native gels containing 0.25X TBM buffer (25 mM Tris-HCl pH 8.9 and 22.5 mM borate with 5 mM MgCl_2_) and run at 150 V at 4°C. All quantifications were performed using a phosphor imager. The composition of monomer buffer is 50 mM Na-cacodylate pH 7.5, 250 mM KCl.

### Conformer Assay

Approximately 2 pmol of radiolabeled RNA template was heated at 65°C for 10 min, slowly cooled to 37°C and kept for further 30 min at this temperature in 10 µl of Tris buffer (10 mM Tris-Cl, pH 7.5), TN buffer (0.1 M NaCl, 10 mM Tris-HCl, pH 7.5), TN buffer with 0.1 mM, 1 mM or 5 mM MgCl_2_, TEN buffer (0.1 M NaCl, 10 mM Tris-HCl [pH 7.5], 1 mM EDTA,[pH 8.0]), formamide buffer (95% formamide, 0.025% SDS, 0.025% bromophenol blue, 0.025% xylene cyanol FF, 0.025% ethidium bromide and 0.5 mM EDTA), dimerization buffer 50 mM Na-cacodylate (pH 7.5, 250 mM KCl, 5 mM MgCl_2_) and then analyzed on a 4% non-denaturing polyacrylamide 0.25X TBE gels and run at 120 V at 4°C. For conformer assays, the radiolabeled HIV-1 RNA transcript (∼2pmol) was incubated at above mentioned conditions in 10 µl of TEN buffer with increasing concentration of PNA oligomers and then analyzed in 4% non-denaturing polyacrylamide 0.25X TBE gels.

### Coupled Dimerization and Template-switching Assay

The efficiency of heterodimerization between the donor and acceptor RNA templates was performed by incubating radiolabeled donor transcript with increasing molar ratio of unlabeled acceptor transcript in dimerization buffer and analyzed on 4% native TBM gel. For template-switching assay, Primer GR (5′ GCC CAT ACT ATA TGT TTT AAT 3′) was labeled at 5′ end by incubating at 37°C for 30 min with T4 polynucleotide kinase and (γ-^32^P) ATP in the reaction buffer (50 mM Tris-HCl (pH 7.6), 10 mM MgCl_2_, 5 mM DDT, 1 mM spermidine and 0.1 mM EDTA). The radiolabeled primer (GR) was heated with donor and acceptor transcripts at a molar ratio of 1∶2:10 at 85°C for 2 min and then snap-cooled on ice. RNA (100 nM) with increasing concentration of anti-sense PNA oligomers (1 to 20 pM) were added to 20 µl dimerization buffer and incubated at 65°C for 10 min and then cooled to 37°C and maintained for further 30 min. Reverse transcription was carried out by incubating above reaction mixture at 10 nM of RNA concentration, at 37°C for 30 min in a mixture containing 50 mM Tris-HCl (pH 8.3), 75 mM KCl, 5 mM MgCl_2_, 10 mM dithiothreitol, 100 µM concentrations of each dNTP, and 0.2 U of HIV-1 RT/µl. The reaction was terminated by the addition of 10 mM EDTA. The samples were analyzed by running at 150 V on a 6% denaturing polyacrylamide TBE gels. TBE gel bands were quantified and the template-switching efficiency of each PNAs was calculated.

### Statistical Analysis

Data are expressed as means ±SD. Statistical significance among groups was determined using the Student t test and the differences were considered significant at P<0.05.

## Results

### UV Thermal Melting Studies of PNA: RNA/DNA Duplexes

**Figure 3:**
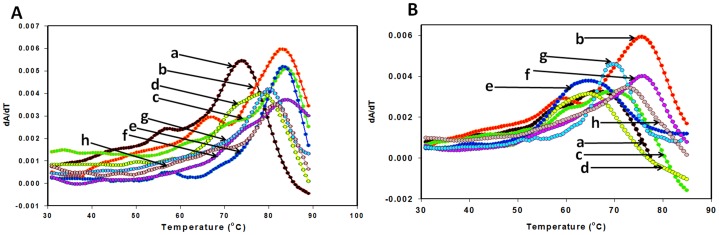
UV T_m_ curves. UV T_m_ curves (A)PNA :RNA antiparallel duplexes of (a)PNA 72, (b)PNA 98, (c) PNA 102,(d) PNA 110, (e) PNA 237,(f) PNA 251,(g) PNA 271, (h) PNA 332, (B) PNA :DNA antiparallel duplexes of (a) PNA 72,(b) PNA 98,(c) PNA 102,(d) PNA 110, (e) PNA 237,(f) PNA 251,(g) PNA 271,(h) PNA 332. 10 mM sodium phosphate buffer, pH 7 containing NaCl (100 mM) and EDTA (0.1 mM) buffer was used for UV T_m_ melting studies.

The thermal melting studies were done to show the binding affinity of synthetic PNA to RNA and DNA. The T_m_ values of different PNAs hybridized with DNA/RNA for antiparallel binding of PNA to corresponding RNA were determined from temperature-dependent UV absorbance and summarized in Table 2. The UV melting temperature data (Table 2) obtained from the melting curves (for UV T_m_ curves Fig. 3, and Fig. S1 & S2) shows that the thermal stability of PNA: RNA duplexes were significantly higher than the corresponding PNA:DNA duplexes. In the case of PNA:RNAduplex, the PNA 237 and 271 showed higher stabilizing effect, having a T_m_ value 83.5°C (Δ T_m_ 19.5°C) and 80.5°C (Δ T_m_ 16.9°C) with RNA. Similarly the other PNAs namely, 98, 102, 251 and 332 also showed thermal stability with corresponding RNA i.e.; 83.9°C, 82.8°C, 84.8°C, 81.4°C respectively, than the corresponding PNA: DNA duplexes (PNA 98 -Δ T_m_ 8.7°C; PNA 102 -Δ T_m_ 12.5°C; PNA −251 Δ T_m_ 7.5°C; PNA−271 Δ T_m_ 16.5°C; PNA 332-Δ T_m_ 9.5°C). PNA 73 and PNA 110 also have stabilizing effect with corresponding PNA than the DNA (Fig. 3 and Table 2). The same UV melting experiments were also performed in dimerization buffer with 5 mM MgCl_2,_ and melting temperature found similar to sodium phosphate buffer with little variation (1 to 3°C) in melting temperature of duplexes (data not shown).

**Table 2:** UV Melting temperature (°C) Value of PNA: RNA and PNA: DNA Duplexes*.

No.	PNA	PNA:RNA	PNA:DNA	ΔT_m_(RNA-DNA)
1	PNA 73	73.9	65	8.9
2	PNA 98	83.9	75.2	8.7
3	PNA 102	82.8	70.4	12.5
4	PNA 110	77.7	64.8	12.9
5	PNA 237	83.5	64	19.5
6	PNA 251	84.8	77.3	7.5
7	PNA 271	80.9	64	16.9
8	PNA 332	81.4	72	9.4

* All values are average of at least three experiment. Buffer- 10 mM sodium phosphate buffer, pH 7 containing NaCl (100 mM) and EDTA (0.1 mM).

### Effect of PNA Oligomers on the Dimerization of HIV-1 Leader RNA

**Figure 4:**
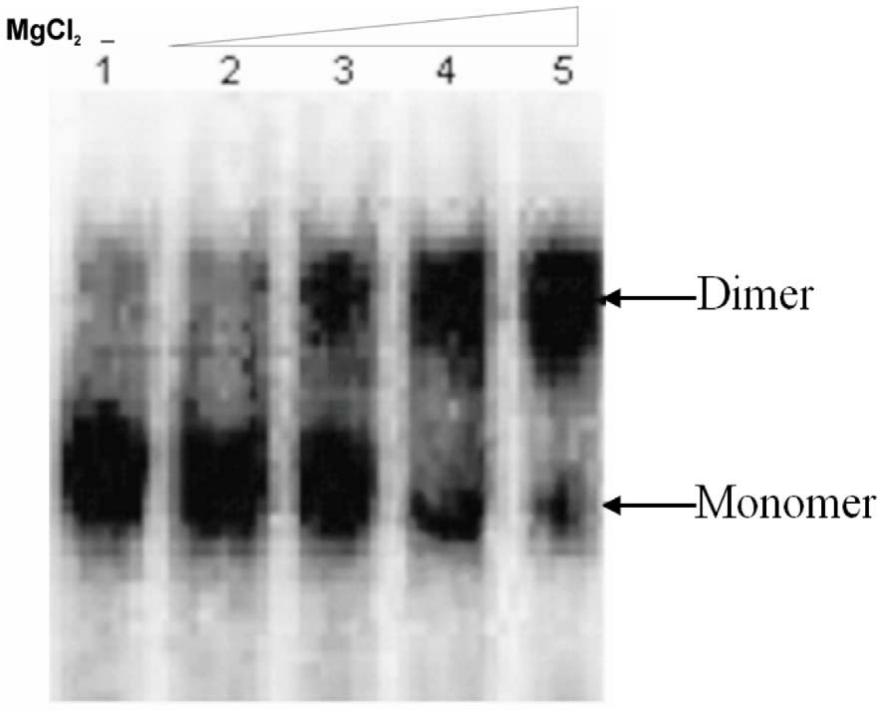
Dimerization efficiency of the HIV-1 RNA (1–447) transcript. The RNA transcripts were incubated in monomer (no MgCl_2_) and dimerization buffer with varying concentration of MgCl_2_. All samples were analyzed on TBM gels. Lane1: monomer buffer, Lane 2: Dimerization buffer with 1 mM MgCl_2_ Lane 3: Dimerization buffer with 2 mM MgCl_2_, Lane 4: Dimerization buffer with 5 mM MgCl_2_, Lane 5: Dimerization buffer with 10 mM MgCl_2._

**Figure 5:**
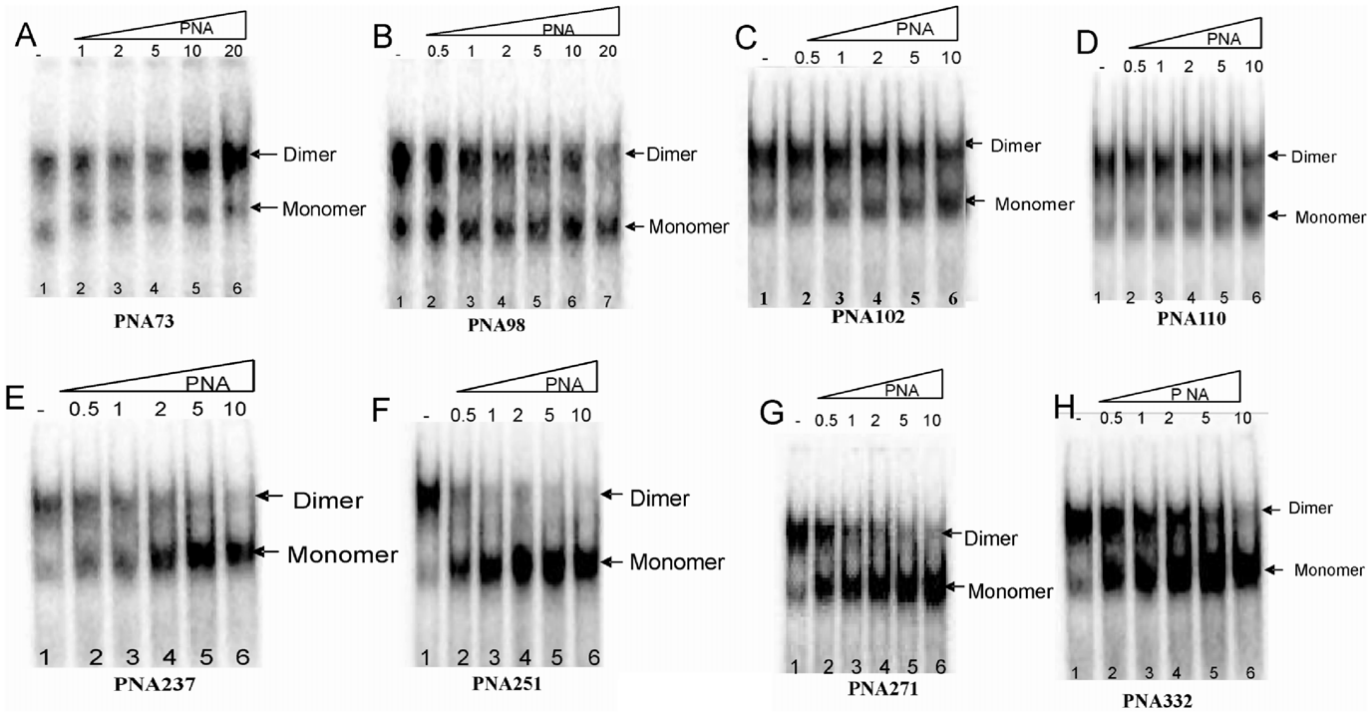
Effects of PNA oligomers on gel mobility of HIV-1 leader RNA (447nts). The RNA transcript was incubated in dimerization buffer (5 mM MgCl_2_) at 65°C in the absence or presence of increasing concentration of PNAs. The samples were analyzed on TBM gels. Each gel has the following loading pattern - Lane 1: Dimerization buffer without PNA oligomer, Lane 2–6: Dimerization buffer with the increasing template ratios, as indicated.

**Figure 6:**
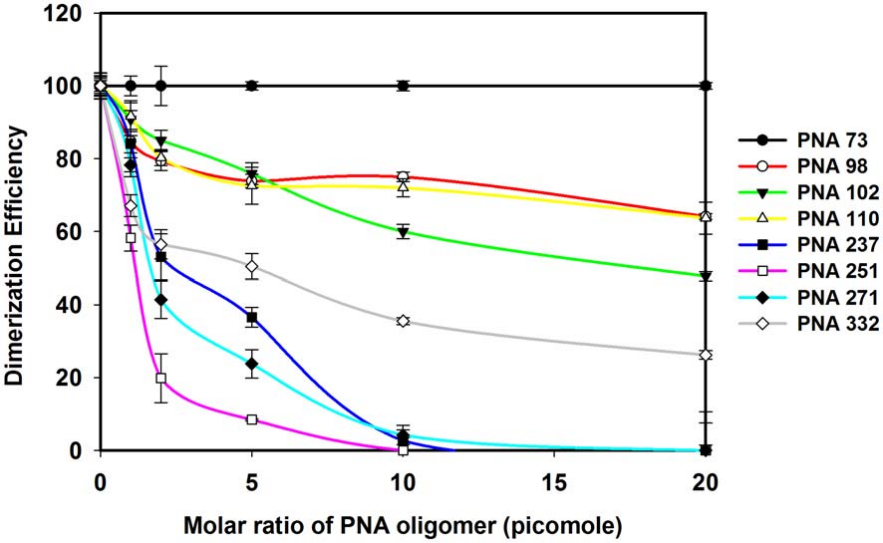
Effects of PNA oligomers on the efficiency of dimerization of HIV-1 RNA transcripts. The samples from dimerization assays were analyzed on the 4% non-denaturing gels and the intensity of monomer and dimer bands on each gel was quantified. The dimerization efficiency was calculated as d/D × 100%, where d is the percentage intensity of dimer with different concentration of PNA and D is the percentage intensity of dimer in the absence of PNAs. Error bars indicate standard deviation from triplicate runs.

To determine the optimal buffer condition for dimerization of HIV-1 RNA (1-447) transcript, RNA was incubated in different buffers then analyzed on TBM (Fig. 4). Based on these results, further dimerization assays were performed by incubating RNA transcripts in dimerization buffer with 5 mM MgCl_2_ at 65°C with varying concentration of PNA oligomers (Fig. 5 & Fig. 6). The results show that the PNA73 increases the RNA dimer formation but only at higher concentrations (10 or 20 fold excess of PNA, Fig. 5A). In contrast, PNA 98 which was designed to target against lower stem of poly A hairpin showed a minimal effect on dimerization at higher concentration (10 and 20 fold excess of PNA, Fig. 5B). PNA251 provided marked reduction in dimerization (∼100% inhibition at 2-fold excess of PNA (Fig. 5F). PNA237&271 also showed a significant decrease in dimerization *in vitro* (Fig. 5E, G). PNA332exhibited ∼80% inhibition at 10 molar excess (Fig. 5H), whereas PNA102 and PNA110 showed a minor inhibition at 5 and 10 molar excess (Fig. 5C, D).

### The Effect of PNAs on the Efficiency of Template Switching between the Donor and Acceptor RNA Templates

**Figure 7:**
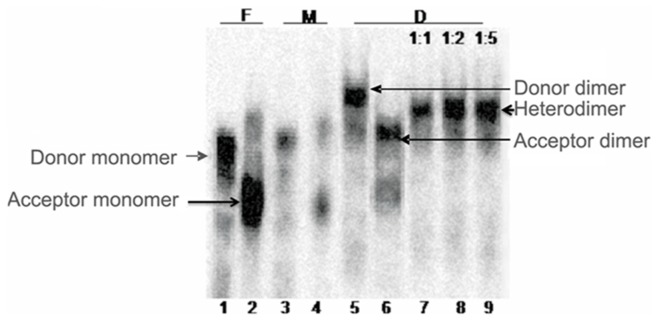
Efficiency of heterodimerization between the acceptor and donor transcripts. The acceptor and donor transcripts were incubated in Formamide, monomer and dimerization buffers, similar to the dimerization assay. The efficiency of heterodimerization between the donor and acceptor was performed by adding molar excess (as indicated at the top of the gel) of unlabelled acceptor transcript to labeled donor transcript under optimal dimerization conditions. All samples were analyzed on the 4% non-denaturing TBE gel. Lane 1: Donor transcript, Lane 2: Acceptor transcript, Lane 3: Donor transcript, Lane 4: Acceptor transcript, Lane 5: Donor transcript, Lane 6: Acceptor transcript, Lane 7–9: Donor transcript with increasing concentration of unlabeled acceptor transcript (1∶1, 1∶2, and 1∶5). F- Formamide buffer, M-monomer buffer and D-dimerization buffer (with 5 mM MgCl_2_).

The schematic representation of both donor and acceptor RNA templates is shown in Fig. 2B. Before performing the template-switching assay, the efficiency of heterodimerization experiment between the donor and acceptor RNA templates was determined. The intensity of heterodimer band was found to increase with the increasing concentration of unlabeled transcript in dimerizaton buffer. (1∶1, 1∶2, 1∶5) (Fig. 7).

**Figure 8:**
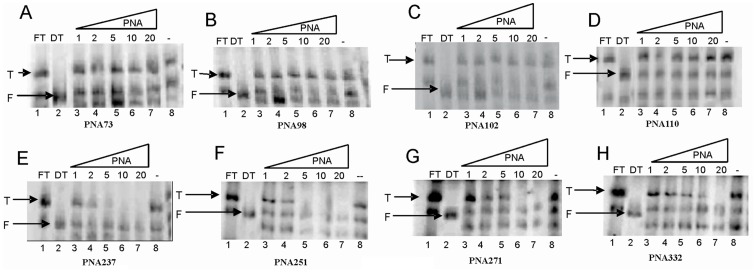
Effect of PNA oligomers on HIV-1 template-switching. Samples were analyzed on the 6% denaturing TBE gel. Each gel has the following loading pattern- Lane 1: HIV-1 leader transcripts including the AUG codon of *gag* gene, Lane 2: Donor transcript, Lane 3–7: Donor and acceptor transcripts with increasing concentration of anti-sense PNAs in molar ratio (with respect to the concentration of donor transcript) as indicated on top of each gel (Reverse transcribed with 0.2 unit of HIV-1RT/µl), Lane 8- Donor and acceptor transcripts without PNA oligo {Reverse transcribed with 0.2 unit of HIV-1RT/µl (from Lane 3 to 8 in each panel )},. FT- Full-length transcript, DT- Donor transcript, T- Template-switched product (Lane 3 to 8), F- Full-length donor product (Lane 3 to 8).

**Figure 9:**
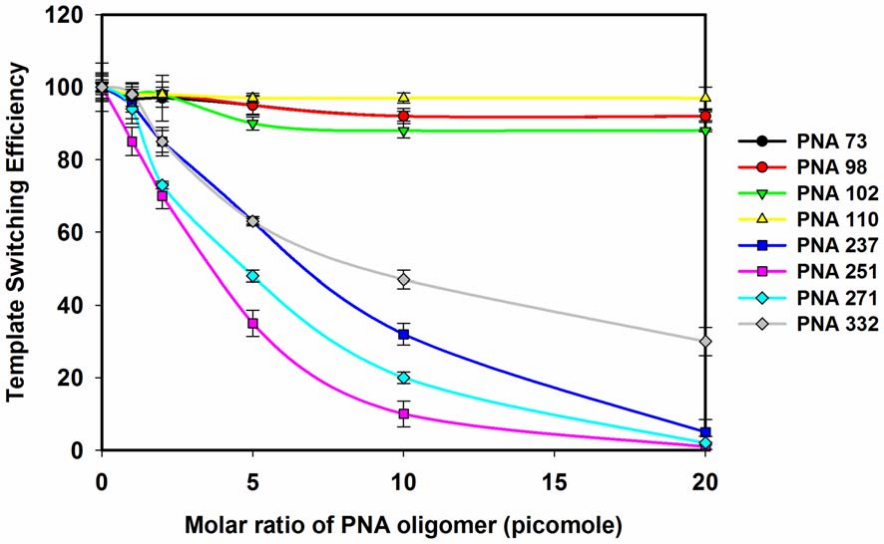
Effects of PNA on the template-switching efficiency between the acceptor and donor HIV-1 RNA templates. The RNA samples from the coupled dimerization-template switching experiments were analyzed on the 6% TBE denaturing gels and the intensity of bands corresponding to the template-switched products and the full-length product of the donor on each gel was quantified The template-switching efficiency for each sample was calculated as t/T × 100%, where ‘t’ is the % intensity of template-switching product in the primer extension reaction with different concentration of PNA oligomer and ‘T’ is the % intensity of the template-switching product in the primer extension reaction without PNA oligomers. Error bars indicate standard deviation of triplicate runs.

The combined dimerization and template-switching assay showed varying degrees of PNA inhibition of dimerization; PNA237, 251 and 271 exhibited an inhibitory effect on template-switching efficiency (Fig. 8, Fig. 9 and Fig. S3).

Maximum inhibition was observed with PNA251, followed by 271 and 237 respectively (Fig. 8 E, F, and G). PNA251 exhibited complete inhibition of template-switching at 20 pM while PNA271 showed 92% inhibition and PNA 237 showed 90% at 20 pM concentration (Fig.9). PNA332 also showed a concentration dependent inhibition of template-switching process showing 60% inhibition at 20 pM (Fig. 8H and 9). However, PNA73, 98, 102,& 110 did not show any effect on the template-switching efficiency (Fig. 8A, B, C, D, Fig 9 and Fig. S3).

### Effect of PNAs on the Equilibrium between LDI-BMH Conformations of HIV-1 Leader RNA

**Figure 10:**
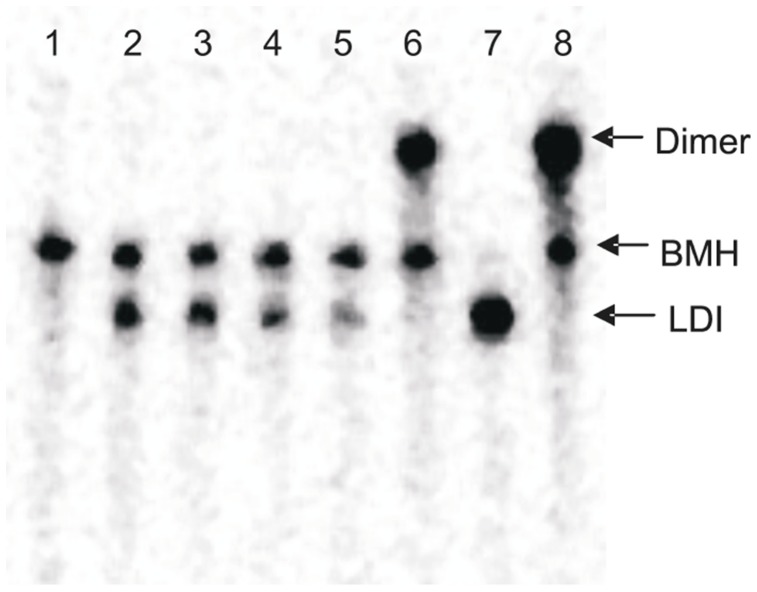
Mobility of HIV-1 leader RNA (447nts) in different buffers. The RNA transcripts were incubated in different buffer conditions and were analyzed on 4% non-denaturing TBE gel. Lane 1: Formamide buffer, Lane 2: Tris-Cl buffer, Lane 3: TN buffer, Lane 4: TN buffer with 0.1 mM MgCl_2_, Lane 5: TN buffer with 1.0 mM MgCl_2_, Lane 6: TN buffer with 5 mM MgCl_2_, Lane 7: TEN buffer and Lane 8: Dimerization buffer with 5 mM MgCl_2_.

**Figure 11:**
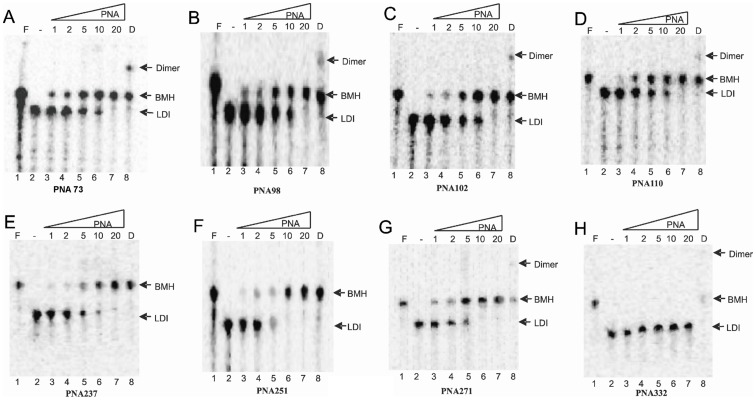
RNA (447nts) in presence of increasing concentration of PNA oligomers. The radiolabeled HIV-1 leader transcript was incubated in TEN buffer with increasing concentration of respective PNA as well as in formamide and dimerization buffer. The samples were analyzed on non-denaturing TBE gels. Each gel has the same loading pattern. Lane 1: Formamide buffer, Lane 2: TEN buffer without PNA oligomer, Lane 3–7: TEN buffer with the increasing concentration of PNA (in molar ratio) as indicated on top of each gel, Lane 8: Dimerization buffer. F – Formamide buffer, D – Dimerization buffer.

**Figure 12:**
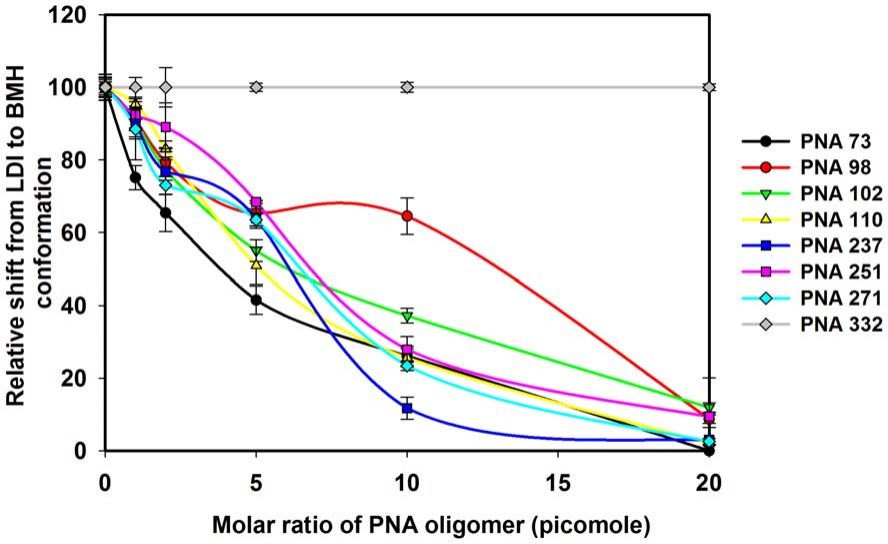
PNA effect on the equilibrium between the LDI and BMH conformations of HIV-1 leader RNA. The RNA samples from conformer assays were analyzed on the non-denaturing gels and the intensity of bands corresponding to the LDI and BMH conformers on each gel was quantified. The relative shift from LDI to BMH conformation was calculated as e/E × 100%, where ‘e’ is the percentage intensity of LDI conformer in TEN buffer with different concentration of PNA oligomer and ‘E’ is the percentage intensity of LDI conformer in TEN buffer without PNA oligomers. Error bars indicate standard deviation of triplicate runs.

The results showed that thermodynamically stable LDI conformer was obtained only in TEN buffer while both LDI and BMH were observed in varying percentages in Tris-Cl, TN buffer (Fig. 10). The dimers formed by RNA transcripts were observed in dimerization and TN buffer containing 5 mM MgCl_2_. These results indicate that all the PNA oligomers (Table1), except PNA332 denature the LDI conformation and shift the equilibrium towards the BMH conformation (Fig. 11 & Fig.12). PNA102 & 110 targeted against the U5 strand of U5-AUG duplex structure, denatured the LDI conformation and shifted the equilibrium towards the BMH conformation (Fig. 11C, D), whereas PNA332 did not shift the equilibrium (Fig. 11H). PNAs targeted against the DIS stem-loop structure, i.e. PNA237, 251 &271, showed the same pattern, shifting the equilibrium towards the BMH conformation (Fig. 11E, F,G). PNAs targeted against poly A hairpin i.e.; PNA73 &98 shifted the equilibrium towards the BMH conformation (Fig. 11A & B).

## Discussion

The antisense oligonucleotide offers an attractive concept, based on the complementarity to a unique sequence on the RNA target, leading to the interference with a given function by “hybridization arrest” [43], [51], [52]. Previous studies demonstrated that sense and antisense oligonucleotides inhibit *in vitro* dimerization of viral RNA, offering possible tools to block dimerization of genomic RNA *in vivo* [43]. Zhang et al. (1997) reported that antisense DNAs complementary to downstream region of the SD site hybridize with the dimer form of HIV-1 RNA, thereby dissociating the dimer. However, antisense DNAs complementary to the region upstream from the SD site did not hybridize with the dimer [53]. In continuation to above studies, we have designed few PNA oligomer as an antisense and demonstrated that PNA: RNA mixmers enhance the inhibition of HIV-1 genome dimerization in comparison to any DNA or RNA antisense sequences. To prove the binding of PNA oligomer to DNA the melting experiments were performed and their results suggested that all 8 PNAs (Table 1) have higher thermal stability with corresponding RNA as compared to DNA (Table 2, Fig. 3). The PNA73: RNA duplex had lowest GC content (5 GC pairing), a reason behind the lowest T_m_ in comparison to other PNA:RNA duplexes, having higher GC content (at least 8 or more GC pairing).Although we cannot correlate the melting temperature of oligomers to their effect on conformational switching leading to inhibition of dimerization but, thermal stability measurements of duplexes demonstrate the target selectivity of PNA to RNA over DNA, showcasing PNA an efficient nucleic acids based therapeutic agent.

PNA73 & 98, target the exposed loop and lower stem of poly A hairpin respectively, destabilized LDI conformation at higher concentration (up to 20 molar excess) (Fig. 11A, B). An interaction was observed between PolyA and DIS motifs in LDI conformation [27]. Binding of PNA 73 & 98 prevent the association of DIS and polyA motifs and force RNA to shift towards BMH conformation which possesses exposed DIS hairpin structure. These results are consistent with the result obtained in previous study, in which antisense oligonucleotides that target the poly A domain trigger the LDI to BMH rearrangement [45]. We observed negligible effect on dimerization efficiency of transcripts even at higher molar concentration of PNA 98. This was attributable to limited accessibility of lower stem of polyA hairpin, hence the negligible destabilization of BMH conformation was observed. No effect was observed on the template-switching efficiency by these two PNAs (Fig. 8A, B & 9), suggesting that the PolyA hairpin might not be involved in template-switching directly.

PNA237, 251 & 271 targeted against the stem and apical loop regions of DIS/SL1 hairpin showed a shift of equilibrium towards the metastable BMH conformation (Fig. 11E, F, G &12). One possible reason for this shift might be due to destabilization of LDI conformation. PNA251, which annealed with the six palindromic nucleotides, was observed to be the most potent one to inhibit dimerization. Therefore, PNA251 was likely to block the initiation of a loop-loop kissing structure (a key step in the dimerization process) with high affinity and specificity. This was consistent with a previous study, which demonstrated that the oligonucleotide directed against the DIS (GCGCGC in HIV-1 Type A) lead to the complete inhibition of dimerization of viral RNA under *in vitro* conditions [43], [44]. PNA237 & 271 targeted against the lower stem of DIS hairpin showed a significant reduction in the dimerization efficiency. These results support previous findings, in which standard oligonucleotides (ODNs) and Locked Nucleic Acids (LNA) containing ODNs were used as antisense agents for the inhibition of dimerization of HIV-1 RNA [43], [54]. Hence, the inhibition of *in vitro* dimerization of HIV-1 RNA by these three PNAs was observed to be in the following order: PNA251>PNA271>PNA237 (Fig. 6).

The coupled dimerization and template-switching assay demonstrated that PNA237, 251 and 271, targeted against the DIS hairpin, reduced template-switching and dimerization with similar potencies. Inhibition is presumably due to annealing of these PNAs to their complementary sequences within the DIS hairpin region and blockage of initiation and maturation of loop-loop kissing complex, which was necessary for the formation of a stable dimer complex between two RNA molecules and consequently inhibit the strand transfer during reverse transcription. Therefore, our results confirmed a direct relationship between the efficiency of template-switching and the stability of DIS-mediated kissing-loop complex [55]. PNA 102 &110 were targeted against the U5 and PNA332 against AUG regions, with the objective of analyzing their effect on the equilibrium between LDI and BMH, dimerization efficiency and template switching efficiency. PNA332 (complementary to the Gag region) showed maximum inhibition (∼80% inhibition at 10 molar excess of this PNA) of dimerization of the RNA transcripts and this might be due to complete denaturation of U5-AUG duplex structure. The other possible explanation might be, hybridization of PNA332 to its complementary sequence, facilitate the interaction between Poly(A) and DIS sequences and helped in the shifting of the equilibrium from BMH to LDI (a dimerization incompetent) conformation. It also showed a concentration dependent inhibition of template-switching process (Fig. 9).The previous studies also showed that the metastable BMH folding was facilitated by stabilization of the U5-AUG interaction [13], [56]. The inhibition of dimerization and strand transfer by PNA 332 is consistent with destabilization of AUG region of BMH conformation. These results showed that the start (AUG) codon region of the Gag ORF is not actively involved in the stabilization of the LDI conformation. PNA 102 &110, which target the U5 strand, denatured the LDI conformation and shifted the equilibrium towards the BMH conformation (Fig. 11C, D). Several studies were published suggesting different structural models of HIV-1,5′-leader RNA. Recently, an in vitro NMR structural model was reported proposing U5-AUG duplex formation. U5:AUG formation promotes dimerization by displacing and exposing a dimer promoting hairpin structure [57]. It was reported that U5:AUG duplex was found exclusively in BMH confirmation and the metastable BMH conformer was facilitated by stabilization of the U5-AUG interaction. However, our results suggested that at increasing concentration of PNA 102&110, the LDI conformation got stabilized to BMH conformation. The annealing of PNA102 &110 to complementary nucleotides in the U5 region showed only a minor reduction in the dimerization efficiency *in vitro* (Fig. 6). It was reported that stabilization of the U5-AUG duplex, via mutation stimulates dimerization of partial HIV-1 transcripts, suggesting that artificial duplex formation promotes RNA dimerization [53]. If U5-AUG duplex exists, reduced dimerization in the presence of targeting PNAs is anticipated, and our failure to make this observation suggests that U5 region of duplex was either inaccessible to PNA recognition, due to steric hindrance in the structure or in the presence of PNA 102 & 110 a certain percentage of LDI form was available at equilibrium position to bind with these PNAs. Palliart *et al.* also do not observe distant base pairing between the nucleotides 105 -115(U5) and 331 -344 (AUG) duplex in infected cells *in vivo*, which clearly support our results (14). Nucleotide resolution SHAPE data also did not confirm the *in vitro* models of RNA and suggested that dimerization in mature viruses occur through formation of intermolecular duplexes (28), neither through TAR stem nor through intramolecular base pairing in DIS stem. Sakuragi *et. al.* proposed a novel HIV-1 dimerization linkage sequence (DLS) structural model, showing a thermodynamically stable pseudoknot like structure having a unique shape with new stem like duplex (GCGUC –GACGC) and alongwith it U5 : AUG duplex sunken in this structure [58]. This new model once again showed a sterically hindered position of U5:AUG duplex.

Based on all above mentioned results, we may conclude that HIV-1 leader RNA adopt two alternative conformations i.e.; LDI and BMH and the switch between these two conformations regulates some of the leader- encoded functions such as RNA dimerization [59], [60] and template-switching during reverse transcription [61]. Thus, our results indicate a close correlation between the LDI-BMH equilibrium, dimerization and template-switching processes of HIV-1 RNA. Although Weeks *et al.* and Palliart *et al.* did not observe long distance interacting conformers in the RNA isolated from the virion but they also did not rule out possibility of a preform which melt to produce dimerization competent form. The disappearance of long distance interacting conformers may be attributed to the more pronounced BMH form which is dimeric and packaging competent, obtained in in vivo conditions, though the former form cannot be neglected in the minor population or for short period of time. The ex vivo structure of 5′ UTR is not thermodynamically stable which shows that LDI conformer is kinetically unfavorable for reverse transcription of RNA (14). We also demonstrated that SL1 or DIS is necessary for initiation and maturation or stabilization of loop-loop kissing complex during the dimerization process and hence template switching process. We may conclude that PNAs can be used as a tool to unravel and correlate the various conformationally controlled viral processes, thereby providing a potent antisense molecule for efficient attenuation of emergence of virulent HIV strains.

## Supporting Information

Figure S1 Melting profiles of PNA with corresponding RNA and DNA duplexes. **Panel A**. **A1**: UV absorbance of PNA 73 with antiparallel RNA, **A2** corresponding first derivative, **A3** UV absorbance of PNA 73 with antiparallel DNA, **A4** corresponding first derivative. **Panel B**. **B1**: UV absorbance of PNA 98 with antiparallel RNA, **B2** corresponding first derivative, **B3** UV absorbance of PNA 98 with with antiparallel DNA, **B4** corresponding first derivative. **Panel C**. **C1**: UV absorbance of PNA 102 with antiparallel RNA, **C2** corresponding first derivative, **C3** UV absorbance of PNA 102 with antiparallel DNA, **C4** corresponding first derivative. **Panel D**. **D1**: UV absorbance of PNA 110 with antiparallel RNA, **D2** corresponding first derivative, **D3** UV absorbance of PNA 110 with antiparallel DNA, **D4** corresponding first derivative.
(TIF)

Figure S2 Melting profiles of PNA with corresponding RNA and DNA duplexes. Panel A. A1: UV absorbance of PNA 237 with antiparallel RNA, A2 corresponding first derivative, A3 UV absorbance of PNA 237 with antiparallel DNA, A4 corresponding first derivative. Panel B. B1: UV absorbance of PNA 251 with antiparallel RNA, B2 corresponding first derivative, B3 UV absorbance of PNA 251with antiparallel DNA, B4 corresponding first derivative. Panel C. C1: UV absorbance of PNA 271 with antiparallel RNA, C2 corresponding first derivative, C3 UV absorbance of PNA 271 with antiparallel DNA, C4 corresponding first derivative. Panel D. D1: UV absorbance of PNA 332 with antiparallel RNA, D2 corresponding first derivative, D3 UV absorbance of PNA 332 with antiparallel DNA, D4 corresponding first derivative.
(TIF)

Figure S3 Gel electrophoresis to evaluate the effect of PNA oligomers on the template-switching efficiency between the acceptor and donor HIV-1 transcripts: Acceptor, donor transcripts and radiolabeled GR primer (anneals to the donor transcript) were incubated in the dimerization buffer with increasing molar excess of respective PNA oligomer, as indicated at the top of each gel. This reaction mixture was used for the next template-switching efficiency assay, for details see Materials and Methods. Samples were analyzed on the 6% denaturing TBE gel. Each gel picture having the following loading pattern. Lane1: HIV-1 leader transcripts including the AUG codon of gag gene, Lane2- Donor transcript, Lane3-7- Donor and acceptor transcripts with increasing concentration of anti-sense PNA in molar ratio (with respect to the concentration of donor transcript) as indicated on top of gel, Lane8- Donor and acceptor transcripts without PNA oligo. FT- Full length transcript, DT- Donor transcript, T- Template-switched product, F- Full-length donor product.
(TIF)
