# Supplementary figures and images for: Inhibition of 5′-UTR RNA Conformational Switching in HIV-1 Using Antisense PNAs
Source: PLoS One. 2012 Nov 12;7(11):e49310. doi: 10.1371/journal.pone.0049310 (PMC3495914; doi:10.1371/journal.pone.0049310)

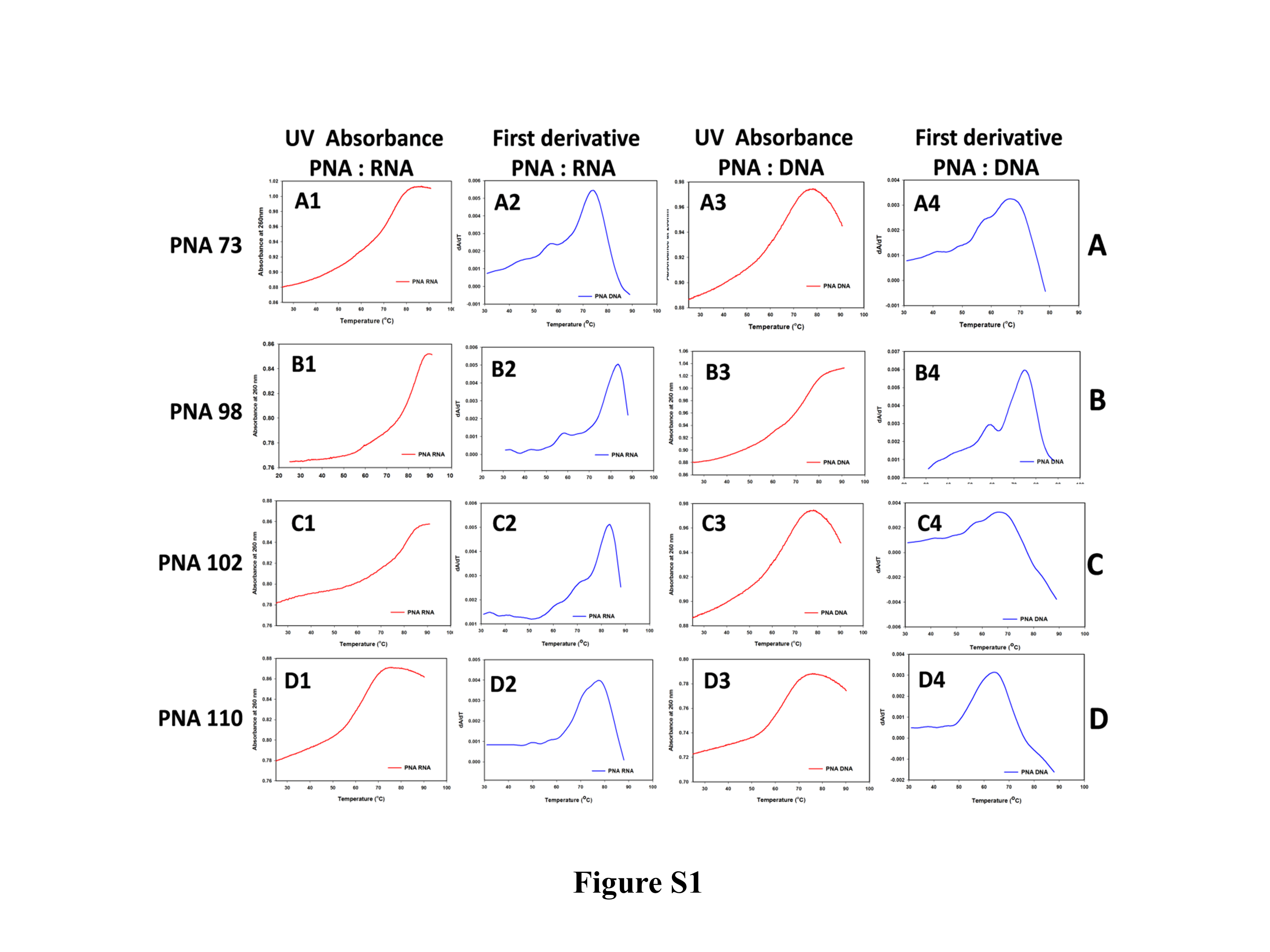

Supplement: Figure S1 — Melting profiles of PNA with corresponding RNA and DNA duplexes. Panel A. A1: UV absorbance of PNA 73 with antiparallel RNA, A2 corresponding first derivative, A3 UV absorbance of PNA 73 with antiparallel DNA, A4 corresponding first derivative. Panel B. B1: UV absorbance of PNA 98 with antiparallel RNA, B2 corresponding first derivative, B3 UV absorbance of PNA 98 with with antiparallel DNA, B4 corresponding first derivative. Panel C. C1: UV absorbance of PNA 102 with antiparallel RNA, C2 corresponding first derivative, C3 UV absorbance of PNA 102 with antiparallel DNA, C4 corresponding first derivative. Panel D. D1: UV absorbance of PNA 110 with antiparallel RNA, D2 corresponding first derivative, D3 UV absorbance of PNA 110 with antiparallel DNA, D4 corresponding first derivative. (TIF) [file pone.0049310.s001.tif]

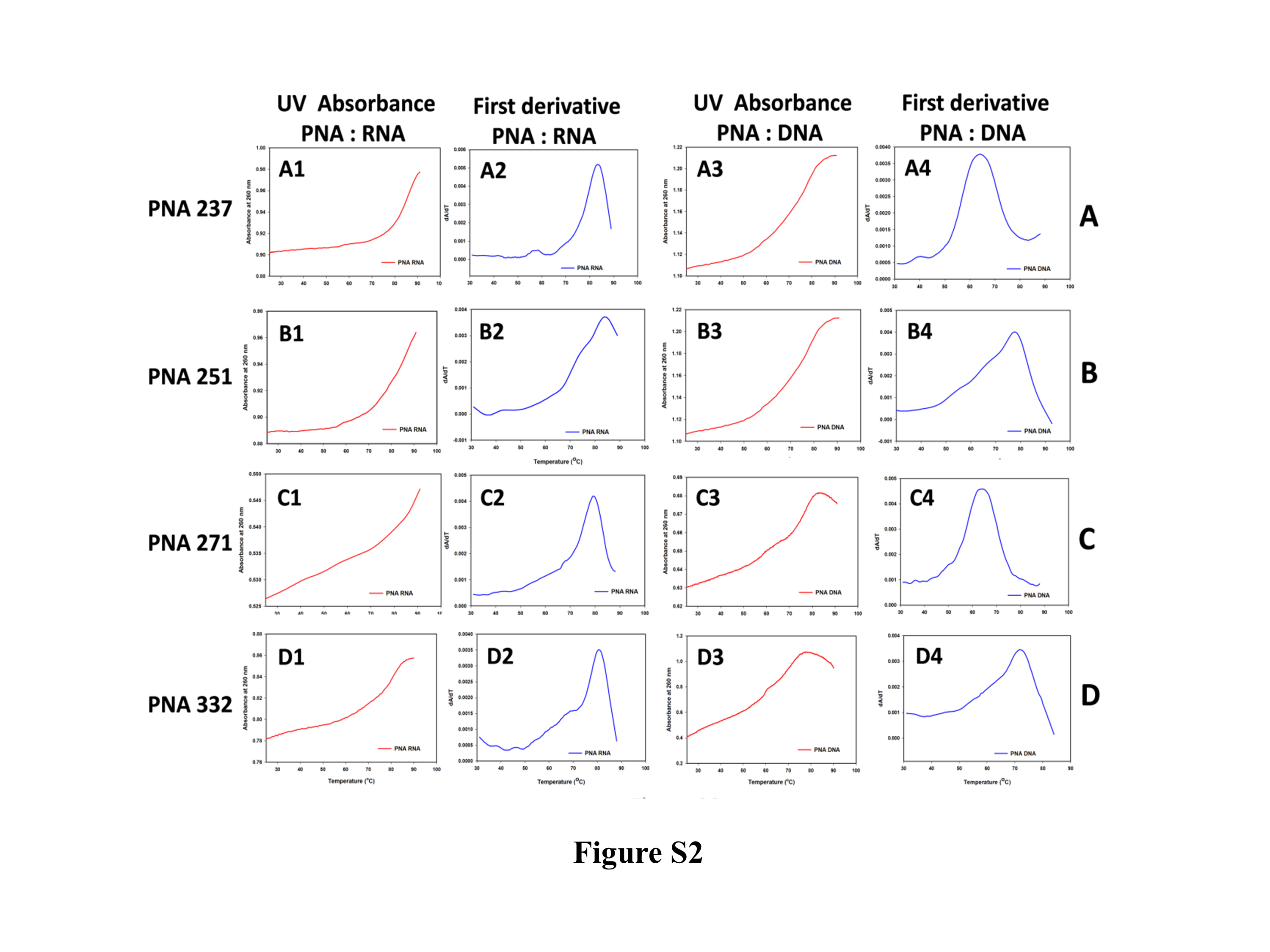

Supplement: Figure S2 — Melting profiles of PNA with corresponding RNA and DNA duplexes. Panel A. A1: UV absorbance of PNA 237 with antiparallel RNA, A2 corresponding first derivative, A3 UV absorbance of PNA 237 with antiparallel DNA, A4 corresponding first derivative. Panel B. B1: UV absorbance of PNA 251 with antiparallel RNA, B2 corresponding first derivative, B3 UV absorbance of PNA 251with antiparallel DNA, B4 corresponding first derivative. Panel C. C1: UV absorbance of PNA 271 with antiparallel RNA, C2 corresponding first derivative, C3 UV absorbance of PNA 271 with antiparallel DNA, C4 corresponding first derivative. Panel D. D1: UV absorbance of PNA 332 with antiparallel RNA, D2 corresponding first derivative, D3 UV absorbance of PNA 332 with antiparallel DNA, D4 corresponding first derivative. (TIF) [file pone.0049310.s002.tif]
